# Scale-out of a community-based behavioral intervention for childhood obesity: pilot implementation evaluation

**DOI:** 10.1186/s12889-018-5403-z

**Published:** 2018-04-13

**Authors:** William J. Heerman, David Schludnt, Dawn Harris, Leah Teeters, Rachel Apple, Shari L. Barkin

**Affiliations:** 1 Division of General Pediatrics, Vanderbilt University Medical Center, 2146 Belcourt Ave, Nashville, 37212 TN USA; 2 Department of Psychology, Vanderbilt University, 2301 Vanderbilt Place, Nashville, 37240 TN USA; 3 School of Education, University of Colorado Boulder, 249 UCB, Boulder, 80309 Colorado USA; 4 Division of Internal Medicine and Pediatrics, Vanderbilt University Medical Center, 2146 Belcourt Ave, Nashville, 37212 TN USA

**Keywords:** Childhood obesity, Intervention scale-out, Community implementation

## Abstract

**Background:**

Expanding the use of evidence-based behavioral interventions in community settings has met with limited success in various health outcomes as fidelity and dose of clinical interventions are often diluted when translated to communities. We conducted a pilot implementation study to examine adoption of the rigorously evaluated Healthier Families Program by Parks and Recreation centers in 3 cities across the country (MI, GA, NV) with diverse socio-cultural environments.

**Methods:**

Using the RE-AIM framework, we evaluated the program both quantitatively (pre/post surveys of health behavior change; attendance & fidelity) and qualitatively (interviews with Parks and Recreation staff and participants following the program).

**Results:**

The 3 partner sites recruited a total of 26 parent-child pairs. *REACH:* Among the 24 participants who completed pre/post surveys, 62.5% were 25–34 years old, and average child age was 3.6 (SD 0.7) years. The distribution of self-reported race/ethnicity was 54% non-Hispanic White, 38% non-Hispanic Black, and 8% Latino. *EFFECTIVENESS:* Qualitative interviews with participants demonstrated increased use of the built environment for physical activity and continued use of key strategies for health behavior change. *ADOPTION:* Three of five (60%) collaborating sites proceeded with implementation of the program. *IMPLEMENTATION:* The average attendance for the 12-week program was 7.6 (SD 3.9) sessions, with 71% attending > 50% of sessions. Average fidelity for the 12 weekly sessions was 25.2 (SD 1.2; possible range 9–27). *MAINTENANCE:* All 3 partner sites continued offering the program after grant funding was complete.

**Conclusions:**

This pilot is among the first attempts to scale-out an evidence-based childhood obesity intervention in community Parks and Recreation centers. While this pilot was not intended to confirm the efficacy of the original trial on Body Mass Index (BMI) reduction, the effective and sustained behavior change among a geographically and ethnically diverse population with high attendance and fidelity demonstrates an effective approach on which to base future large-scale implementation efforts to reduce childhood obesity in community settings.

**Electronic supplementary material:**

The online version of this article (10.1186/s12889-018-5403-z) contains supplementary material, which is available to authorized users.

## Background

Implementation of evidence-based childhood obesity interventions is an important component of combating obesity and its long-term health consequences. Over the last two decades there have been over 300 randomized controlled trials to prevent or treat childhood obesity [1]. While some of these trials have demonstrated efficacy for improving child body mass index (BMI), the generalizability of results and sustainability of these programs are difficult to assess [2–7]. Furthermore, attempts to replicate these programs in other settings has proven particularly challenging. Aarons et al., have recently described these efforts as “scale-out” interventions, where researchers employ the “deliberate use of strategies to implement, test, improve, and sustain an evidence based intervention as it is delivered to new populations and/or through new delivery systems that differ from those in effectiveness trials.” [8] With the burden of childhood obesity now affecting 17% of children in the United States [9], the future of obesity research needs to include rigorous methods for “scaling-out” efficacious studies into real-world contexts and evaluating their impact [10].

Community-based implementation research is an emerging field that focuses on how efficacious interventions should be modified so that similar results can be achieved in different settings with more diverse populations [10]. In their qualitative evaluation of 19 community-based obesity programs in Missouri, Dreisinger et al. developed a framework of individual, organizational, community, and intervention factors that are critical for widespread obesity program distribution [11]. In addition, we have learned from other effective health-behavior change programs, such as the Diabetes Prevention Program (DPP), that to translate effective interventions into a real-world settings requires: community collaboration, adaptation that contains costs while maintaining fidelity, and considerations for maintenance [12].

Despite the identification of these contextual factors required for successful community-based implementation, programs like the DPP have met with more limited success when translated into community settings among traditionally under-represented populations [13]. Very few obesity prevention programs have even attempted scale-out or replication in different community settings, and systematic implementation research is needed to build strategies for translating research into community-based interventions that will effectively improve public health [14, 15]. Thus, the field is ripe for the application of community-based scale-out principles in the service of extending efficacious childhood obesity interventions into a broader range of communities.

This report details the pilot scale-out implementation efforts of the Healthier Families Program. Healthier Families (*Salud con la Familia)* was developed in 2009, and a randomized controlled trial (RCT) demonstrated efficacy at reducing child BMI among a sample of 106 Latino parent-child (ages 3–6) pairs [16]. The intervention was a 12-week family-based skills building intervention conducted in local Parks and Recreation centers in Nashville, TN. The intervention resulted in significant differences in BMI trajectories, comparing the intervention to the control group. In 1-year follow-up, intervention participants were almost four-times more likely to use their Parks and Recreation facilities for physical activity (per self-report) than control group families who had the same geographic access to Parks and Recreation facilities. This finding demonstrated sustainability of healthy behaviors following the intensive behavioral intervention. While the Healthier Families Program demonstrated efficacy in the original trial, it is not clear if it can be successfully scaled-out for a more wide-scale adoption by additional Parks and Recreation centers, especially in different social, cultural, and economic contexts.

In a prior report, we presented the systematic process undertaken to adapt the Healthier Families Program for populations in different socio-cultural contexts [17]. In this manuscript, we assess scale-out implementation of the program when delivered to different populations but through the same public infrastructure of Parks and Recreation departments. We used a mixed-methods approach to evaluate program implementation using all elements of RE-AIM (Reach, Effectiveness, Adoption, Implementation, Maintenance), a robust framework for evaluating the implementation of community-based behavioral interventions [18–20]. RE-AIM has been used in multiple contexts to provide a critical evaluation of the strengths and weaknesses of both individual-level and organizational-level domains, and when used most effectively incorporates both quantitative and qualitative data [21]. The explicit purpose of RE-AIM is to evaluate the public health impact of efficacious interventions in real-word settings [22], and our corresponding goals in this pilot study were simultaneously building the evidence base for childhood obesity prevention and developing best practices for the scale-out of community-based interventions more broadly.

## Methods

### Intervention

The Healthier Families Program is a family-based obesity prevention program for pre-school aged children and the parent(s)/caregiver(s). Weekly sessions are designed to build skills for parent-child pairs to improve health behaviors, including choosing healthy foods, selecting healthy snacks and drinks, grocery shopping, portioning food and drink, creating a fitness home, being an active family together, using media wisely, engaging as a family, and creating healthy sleep habits.

The process of translating the Healthier Families Program included adapting program structure and processes and training interventionists to deliver the content with high fidelity at 3 Parks and Recreation centers with geographic and socioeconomic variation. The final program that was delivered maintained the essential structure and content of the original intervention, consisting of a 12-week intervention, in which weekly sessions lasted 60 min. Adhering to the original program, each session consisted of goal-setting, group problem solving, an active, family-centered skills building component with both parents and preschool age children, and an interactive didactic learning section. Adaptations primarily focused on logistical issues to achieve successful implementation.

Interventionists were initially certified after attending a two-day face-to-face training and completing online learning modules that included general information on childhood obesity, effective facilitation skills, the core structure and processes for each module, and specific step-by-step instructions for each of the 12 sessions. Certification also involved video recording a practice session and submitting it for a fidelity check that was completed by trained health coaches using a detailed, behaviorally anchored checklist (Additional file 1). Two sites trained local staff as interventionists and one trained an independent fitness consultant.

### Participants

Each partner site was responsible for recruitment of 10 families from local communities using in-person, face-to-face, and email approaches. Inclusion criteria for families included parents (ages ≥18 years) and children (ages 3–5 years), English speaking, and a commitment to participate in the 12-week program. Exclusion criteria for families included no telephone contact, or a parent/child medical illness that would prevent participation in regular exercise.

### Data collection

Assessment of the intervention was based on the RE-AIM framework using suggested measures of each domain (Table 1) [18, 22], including key-informant interviews and surveys from local Parks and Recreation staff and program participants. *Reach* is defined as the size and community representativeness of the participants enrolled in an intervention program, and was measured via self-reported participant demographic characteristics, which were compared to U.S. Census data. *Effectiveness* is the impact of an intervention on outcomes, which was assessed via qualitative interviews with participating parents, described below. *Adoption* refers to the number of organizations or settings that choose to implement a program, and quantitative and qualitative data on adoption were obtained from interviews with Parks and Recreation staff and administrators. *Implementation* refers to how well the community implementation of the program (fidelity) corresponds to the design and intent of the original program and how the program was adapted to fit local needs, which we assessed via a fidelity checklist for video-recorded intervention sessions. *Maintenance* refers to the extent to which changes in individual behavior are maintained, which we evaluated using qualitative interviews with participants [23]. We do not present attendance or fidelity data stratified by site at the request of our local Parks and Recreation partners, though there were no differences in either attendance or fidelity across sites.

**Table 1:** Measurement of RE-AIM constructs

RE-AIM element	Definition	Data sources
Reach	• The extent to which populations at risk for obesity participated in the study • The extent to which the population included in the study reflects the target population.	• Demographics from participant surveys prior to study participation • U.S. Census Bureau Data
Effectiveness	• The impact of participation in the program on health behaviors.	• Pre-post survey data from participants, measuring 1) behavior change techniques, 2) strategies for healthy behaviors, and 3) healthy behaviors.
Adoption	• The extent to which target settings (i.e., Parks and Recreation departments) participated in the program	• Pre-program assessment using key-informant interviews and survey data from local Parks and Recreation staff.
Implementation	• The fidelity to the specific components of the intervention protocol	• Completion of on-site and web-based interventionist training • Directly observed fidelity of intervention delivery (video recorded) • Program Attendance
Maintenance—organizational level	• The extent to which organizations sustained the program after grant funding was complete	• Assessment of partner commitment to continue program implementation
Maintenance—individual level	• The extent to which individuals maintained behavior change after the intervention was complete	• Key-informant interviews with program participants 3 months after the study

A quantitative assessment of organizational readiness to adopt the Healthier Families Program was conducted using a modified version of an instrument developed by Helfrich et al. [24], which consisted of 25 items in 6 sub-scales: Mission Alignment (1 item), Leadership Culture (6 items), Leadership Style (6 items), Readiness for Change (4 items), Role Clarity (4 items), and Resources (4 items). Response options for each item were on a 5-point Likert Scale from 1-Strongly Disagree to 5-Strongly Agree. Results are reported as the average score (possible range 1–5) for each sub-domain.

Participant surveys were conducted before and immediately after the 12-week program and were used as indicators of effectiveness for this pilot. This survey consisted of 76 items and measured how frequently participants used behavior change techniques (goal setting, goal monitoring, and meeting goals), how frequently participants used specific strategies for healthy behaviors (100 cal snacks, using food labels, using the plate method, and monitoring screen time), and how frequently children engaged in specific healthy behaviors (< 2 h of TV/day, hours of sleep/day, 5 fruits & vegetables/day, % of plate that is fruits & vegetables, and use of the built environment for physical activity). These items were either coded dichotomously (yes/no) or as the number of times per day or week. A research assistant from the study team conducted semi-structured interviews via telephone with families immediately following the program’s implementation and then 3 months after the program implementation. The research team developed an interview guide that asked questions about the following domains, including probing questions (see Additional file 1): best aspects of the program, areas for improvement, healthy behavior changes that resulted from participation in the program, built environment use that resulted from the program, the success of using behavior change techniques (i.e., goal setting, group problem solving) during the program, barriers to participation, and confidence in having a healthier family because of participating in the program.

All participants who completed the survey or participated in a semi-structured interview signed an informed consent document prior to participation. The Vanderbilt University Medical Center Institutional Review Board approved this study.

### Data analysis

Descriptive statistics (percent or median and inter-quartile range [IQR]) were used to summarize responses to the survey items. While each site received anonymous and individualized reports regarding the responses to survey items at their center, we present them in aggregate here to preserve confidentiality. The survey was administered electronically and data were stored in a secure REDCap database. All data were analyzed using STATA version 14.2 (College Station, TX).

The interviews were audio recorded and the recordings were transcribed by a professional transcription service. Our systematic approach to content analysis involved three stages [25]. First, a coding system was deductively developed based on the moderator’s guide. This coding system was then applied to describe, sort, and analyze the interviewee quotes. After an initial application of the coding system, it was modified to include any missing themes that were not included in the initial scheme. Each quotation could be assigned multiple codes. Coding and data management was done using spreadsheets. Two coders reviewed each transcript and then discussed and resolved any initial differences in coding.

## Results

The quantitative and qualitative data used to evaluate the Healthier Families Program are organized using the RE-AIM framework, and key themes identified through the qualitative analysis are presented in Table 2.

**Table 2:** Qualitative themes inductively identified from key informant interviews, sorted by RE-AIM category (Reach, Effectiveness, Adoption, Implementation, Maintenance)

Theme	RE-AIM category
Increased knowledge of health behaviors	Effectiveness
Equipping families to make behavior change	Effectiveness
Family Engagement	Effectiveness/Maintenance
Community Engagement	Effectiveness/Maintenance
Use of Built Environment	Effectiveness/Implementation
Barriers: Time Constraints	Effectiveness/Implementation
Alignment of Community Priorities	Implementation
Barriers: potential future cost of program	Implementation
Facilitators well-trained to deliver intervention	Implementation
Facilitator professional development skills	Implementation
Barriers: challenging to conduct sessions with children	Implementation
Barriers: logistical challenges for facilitators and families	Implementation
Barriers: continued program funding	Maintenance

### Reach – Size and representativeness of enrolled participants

Parks and Recreation centers invited families in their community with children ages 3–5 years to sign-up for the 12-week program. There were ten slots available at each site (maximum of 30 participants allowed in this pilot implementation project). The final enrollment at each site was *n* = 8, 7 and 9 (Table 3). Of the 26 families that participated, 62.5% of parents were in the 25–34 years old, and average child age was 3.58 (SD 0.67) years. The distribution of self-reported race/ethnicity was 54% non-Hispanic White, 38% non-Hispanic Black/African-American, and 8% Hispanic/Latino. On average, there were 3.1 (SD 1.08) family members less than 18 years old living in the home. The majority of parents were employed full time (62.5%) or were stay-at-home parents (45.8%), and only 1 participant was disabled/unable to work. While 79% of parents had graduated from college or had higher than a college degree, 21% did not graduate from college. There was also variability in household income: 29% of families earning >$75,000 per year, 37.5% earning between $35,000 and $74,999, and 33.3% earning <$35,000 per year. The three sites differed significantly in socio-demographic features.

**Table 3:** Reach of the healthier families program

Population demographics, 2010 [28]	Recruited participants
Michigan (*N* = 8)	
Population: 114,297 • White: 61.2% • Black: 23.7% • Hispanic or Latino: 12.5% • High school graduate of higher: 86.2% • Median household income: $36,054	White, non-Hispanic: 7 or 87.5% Black, non-Hispanic: 3 or 37.5% Hispanic or Latino: 0 High school graduate or higher: 8 or 100% Median household income level: $50.000 to $74,999
Georgia (*N* = 7)	
Population: 691,893 • Black: 54.3% • White: 33.3% • Hispanic or Latino: 9.8% • High school graduate of higher: 88.4% • Median household income: $50,856	White, non-Hispanic: 0 Black, non-Hispanic:7 or 100% Hispanic or Latino: 0 High school graduate or higher: 7 or 100% Median household income level: $10,000 to $19,999
Nevada (*N* = 9)	
Population: 257,729 • White: 76.9% • Hispanic or Latino: 14.9% • High school graduate of higher: 92.5% • Median household income: $64,489	White, non-Hispanic: 7 or 77.8% Black, non-Hispanic: 0 Hispanic or Latino: 2 or 22.2% High school graduate or higher: 88.9% Median household income level: $35,000 to $49,999

Comparison between the population in each of three participating communities with the demographics of recruited participants at baseline. Percentages may not add to 100% as some participants selected more than one race/ethnicity category

A comparison between the sociodemographics of participants who enrolled in the program with the US Census data for their zip code is shown in Table 3. In Michigan, the participants did not represent the state’s Hispanic population, and Whites and Blacks were overrepresented; also, the participating families reported a higher median income and educational attainment than the state. In Georgia, the participating sample was exclusively Black which does not reflect the state’s racial distribution; this may in part explain the lower median income of the sample compared to the state. The Nevada sample was more racially and educationally representative of their state’s population but with lower income. While these pilot samples were not representative of their state’s population, it is uncertain whether they accurately represented the portion of the population that utilizes Parks and Recreation centers for which data were unavailable.

### Effectiveness – Intervention impact on outcomes

**Fig. 1:**
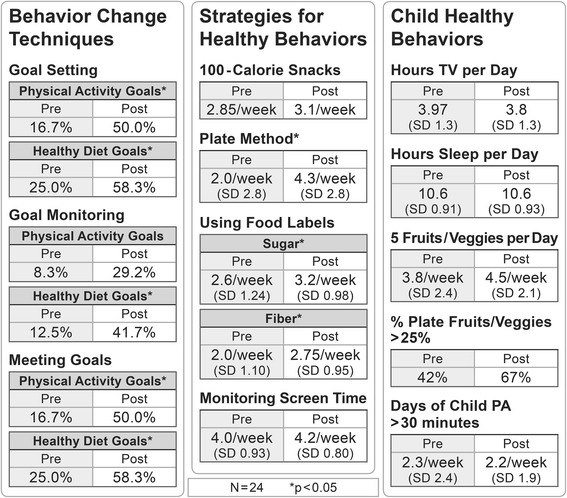
Effectiveness of Healthy Families Intervention among 24 families who completed the post-program survey. Sign-rank tests are used for continuous variables and tests of proportion for percentages. *p < 0.05

The efficacy of the Healthier Families Program has been previously reported [16]. Because this trial was designed as a pilot implementation project, the trial was not powered to evaluate changes in child body mass index. However, surveys conducted before and after the program demonstrated changes in healthy behaviors that are up-stream mediators of BMI change (Fig. 1). Parents reported meeting self-set goals and using behavior change techniques like goal setting and self-monitoring more frequently after the program: before the program 16.7% of parents met physical activity goals, and 25.0% of parents met healthy diet goals. Following the Healthier Families Program, 50.0% (*p* < 0.05) of families met physical activity goals, and 58.3% met healthy diet goals (*p* < 0.05). Parents also reported using strategies for healthy behaviors that were taught during the intervention, including the plate method (*p* < 0.05), using food labels to evaluate sugar content (*p* < 0.05) and fiber content (*p* < 0.05). Parents also reported that their children were engaging in healthier patterns of diet, media use, and physical activity, though not reaching statistical significance.

Qualitative data obtained from 24 participants immediately following the intervention were used to describe changes in important health-related behaviors. For example, families expressed that they had increased knowledge about a variety of topics as a result of participating in the program. Most of the new knowledge that families reported acquiring was related to topics such as healthy sleep, parenting, and media use. Many participants shared that they already knew about healthy foods and physical activity. The program, however, served to encourage and equip families to actually make changes to their physical activity and eating behaviors by providing strategies for engaging their entire family in the change process. When asked about the impact of participating in the program, one participant responded, “I was just telling my husband earlier today, I looked in the mirror and I can already tell I’ve slimmed down. We have eaten less fast food. We were sharing at our last meeting, how people are eating out less, spending more time together. It’s made me more mindful. Things where I was thinking about before, there was no accountability behind it, has made me more mindful.” While another said, “It’s made me more conscious, and making little choices here and there that add up over time. I think that’s when it’s been most beneficial to me, that it’s put it in the forefront of my mind instead of not even thinking about it at all…”.

In general, parents identified the family-engagement aspect of the program as critical to its efficacy. One parent said, “I think it was good for my kids to get that specific instruction because they just seemed more interested. We went to healthy foods class, these are healthy foods, they had some buy-in. It wasn’t just my class, it was theirs too.” Another parent said, “You start the program at the beginning you’re with your kids then your kids leave and you get this great, adult interaction while your kids get to go hang out with friends, and I loved the way it’s set up. It’s set up to be family time, it’s an adult time and then family time again. Something about that was really special.” In response to a question about what made it difficult to meet weekly health behavior goals, one participant said, “All the obstacles like time to plan, illness, holidays, things that just disrupt the pattern of life in general.”

Parents enjoyed having the opportunity to hear ideas about parenting and healthy living from other parents. They seemed to appreciate the opportunity to share ideas and similar life experiences with other parents. One parent said, “I really liked that you got together with other parents. Especially they’re kind of in the same boat you are and just wanting to do good for their families and they’re able to connect and see exactly what they’re doing and see if there’s ways that what they’re doing will help you.” Another mom said, “…the social aspect of it, being around other people that are in a similar stage of life and bouncing ideas off of each other that way where you know that there’s other women going through the same thing.”

When asked specifically about use of their built environment for physical activity, participants indicated increased use of the Parks and Recreation centers as a result of the program. One participant said, “Yes, it did make me want to look more into the activities that are available there so I can sign up the kids. It did interest me, yes.” Some respondents indicated that participating in the program opened their eyes to the availability of the Parks and Recreation centers and the opportunities therein. One participant said, “Actually, it got me interested in looking at the Parks and Rec as an option for going to work out. Before, I had a membership at [local fitness center] and when I realized that they offer some of the same things and some of these classes are free. If it’s not cheaper it’s free. I’m definitely looking at going to a rec center versus paying a membership for a gym. That was one thing that was an eye-opener, like, okay, I didn’t know they had all of these different classes. They also allow you to bring your kids…” There was also a sense that some people were motivated to continue to participate in the activities at the rec centers because other families from the program might be there as well. One participant said, “I didn’t realize there was so much available… then knowing some of the other families were going to do certain things so that we’d all be together again, that is kind of cool too.”

### Adoption—Organizational level

A full description of the determinants of organizational adoption has been reported elsewhere [17]. At the outset of the study four Parks and Recreation centers responded to an email via the National Parks and Recreation list-serv inviting participation, one of which dropped out shortly after the program began due to a change in their building infrastructure and lack of space. We were then able to recruit a fifth recreation center to participate. Thus four Parks and Recreation centers participated in the initial readiness assessment. This assessment included both quantitative evaluation of organizational capacity to change and qualitative key-informant interviews. It was determined that in one site the community had different priorities and less interest in a program aimed at healthy parents and young children. Consequently, three sites fully implemented the program, a 60% adoption rate.

Prior to piloting the program, key informant interviews from community members and organizational leaders demonstrated an eagerness to participate, recognizing close alignment between community priorities and the objectives of the Healthier Families Program. A local recreation center patron shared that the Healthier Families Program, “seems like a very good program to offer in this community. Everyone is very concerned about health and we want to eat right and we want to do right.” Similarly, recreation center staff reported that their team was “really focused on health and wellness and trying to provide that for people in the community.” Another staff member emphasized that this focus was, “not just while [community members] are here with us, but we also consider how do we get them to take that then beyond, and make that part of their lifestyle.”

Following the implementation, Parks and Recreation staff were asked the following questions with a response option of 1–5 (5 = strongly agree): How ready is your center to continue offering the Healthier Families Program? How engaged is leadership in continuing to offer the Healthier Families Program? How much of a priority is the Healthier Families Program in your center? Ratings ranged from 3 to 5 and the means were from 4.3 to 4.7. This shows substantial agreement that the centers felt ready to implement the program, that the leadership was engaged, that the program was a priority, and that it was easy to implement.

Furthermore, participant interviews following the three-month program showed successful adoption. When asked how well-trained their facilitator was, respondents in general seemed to think the facilitators were adequately trained. Several commented that their facilitator was very positive, enthusiastic, and welcoming. Facilitators did not seem to be leaning too heavily on the printed material/reading from a facilitator’s guide. Participants felt they were fairly knowledgeable about the material. Multiple people indicated that they really liked it when their facilitator brought up examples or applications from their own life/own experiences. Several commented on the children’s program facilitators and how much their children enjoyed interacting with these individuals on a weekly basis. Several respondents remarked that while they liked the program, they were not sure what they would be willing to pay to attend a similar class in the future. Many indicated that the fact that the class was free was a major incentive for their participation and/or they would only be willing to pay a nominal fee in the future.

### Implementation – Fidelity to original program intent

Prior to implementation, key adaptations were instituted to contextualize the intervention for each community. These adaptations were made with input from the local recreation center staff in conjunction with the study team, and did not alter the core content of the intervention. Primary adaptations included reducing session time from 90 to 60 min, re-branding for each local site, developing enhanced training modules for new facilitators (emphasizing facilitation and behavior change techniques), implementing a web-based facilitator training program, and providing a snack instead of a meal at the end of each session. Each site made small customizations for printed materials, communication strategies (email) and engagement of children depending on their age and developmental stage.

The average attendance for the 12-week program across all three sites was 7.6 (SD 3.91) sessions, with 43% attending at least 80% of the sessions and 71% attending at least 50% of the sessions. Both families and Parks and Recreation leaders identified time constraints as the biggest barrier to program participation. Families indicated that other activities such as errands, taking children to and from school, travel, and holiday activities, in some cases, prevented them from attending all of the class sessions. No one identified money or finances as barriers to their participation in the Healthier Families Program probably because this pilot program was offered at no-cost to the participants.

Fidelity was assessed by using a checklist to score video recordings of each session submitted by the sites and scored by two independent raters. Average fidelity for the 12 weekly sessions was 25.2 (SD 1.2; possible range 9–27). There were no significant differences in attendance or fidelity across the three sites (data not shown).

When interviewed about implementation following the program, Parks and Recreation leaders reported that implementation was dependent on having the right staff, training them in how to facilitate the program, and allowing them to do their jobs. There were only very minor difficulties with staffing, with most of these comments reflecting upon specific staffing activities or general praise for how well staff did in implementing the program. One respondent said, “I can’t think of anything really specific that didn’t go well. I think we were surprised with how much training was required, but I think, in the end, [staff] and I both realized why that was necessary in making sure that we were doing it the way it needs to be done and fidelity and all of that. Not that it didn’t go well, it was just kind of an interesting journey. Whereas, other programs, we just kind of jump in. Just the commitment, I think, was surprising, but I think well worth it.”

On the other hand, staff were not used to leading sessions in which both parents and young children were present. Staff expressed appreciation of how the Healthier Families Program was able to get adults and children to engage with each other. They saw a number of ways that families could benefit from the parent-child interactions, and from interacting within the structure of the program. In addition, staff reflected on some of the challenges they faced in engaging the families. Several noted that it was a learning process, and the skills involved in engaging families in the process of making change is quite different from the skills involved in classroom teaching. They shared that they got better at doing this over time. One Parks and Recreation leader said, “An ability to connect with families and both kids and adults. That can be really tricky, because they’re two quite different ... To be able to engage both the kids and the grown ups is tricky. I would say being able to lead a discussion I guess more by asking questions, so guiding the discussion rather than leading the discussion. It’s not as much about teaching as it is asking the right questions so people discover it for themselves. That can be really hard if you’re coming at it as a typical teacher or class instructor. It’s not just about giving the information. It’s about helping people find it for themselves.”

While time-intensive for training, working with the Healthier Families Program was perceived as personally beneficial by the Parks and Recreation staff and administrators we interviewed. Staff participants identified a number of ways that participating in the Healthier Families Program allowed their own personal skills to improve. Some felt more confident afterwards, especially in their ability to facilitate group interactions. They felt that specific skills such as listening and getting families to participate also improved. Even the administrators saw a benefit from working with the program as a way to improve skills.

Additionally, Parks and Recreation staff noted some logistical barriers to implementation such as slow internet speeds when uploading video recordings, language issues, dealing with families that brought other children, and finding the ideal time of day to accommodate everyone’s schedule and needs. They also noted that families faced obstacles to participation such as competing activities, scheduling difficulties, family problems, poverty, transportation issues, and staying motivated to attend weekly sessions.

### Maintenance—Individual level

Three months following the intervention, participants completed key-informant interviews, which demonstrated sustained behavior change as a result of the intervention. When asked about any changes made in behaviors as a result of the program, one participant responded that her family was “Exercising more, which we didn’t used to do.” Another responded, “Mainly with the prepping, so we eat healthier now and essentially joined the gym. We work out more. We got more active and involved just because of the accountability of it and just thinking about it, because from the classes, you think about it more.” Another parent responded, “My kids help a lot more with preparing foods now. I think when they get a chance to prepare, they like eating healthier because they worked hard at making it. I think it’s a little bit easier for them to eat things that they wouldn’t normally try just because they made it.” When asked about the most important impact of the program, one participant responded “I think it’s mostly made me be more aware of all of the aspects of having a healthy family. I think before I was really focused on the eating habits, and I guess just helping me realize that there’s more to just eating healthy.” And another responded, “We’ve been exercising a lot more, five times a week now, every week. It’s been like three months now. We’ve been doing that since we ended the program. It’s been pretty good.” One participant commented, “I liked the SMART goals because it just ... You know, it made me realize that it’s not a whole huge change that has to take place at once, but just working on little things day by day, to just be a little bit more conscientious of what you’re doing with food and exercise, and everything between.”

### Maintenance—Organizational level

In key informant interviews with Parks and Recreation staff following the program implementation, the issue of sustaining the program was viewed as a matter of finding funding or partnerships to help underwrite the various costs of the program, with secondary concerns about identifying the right facilitator and finding enough families to fill the program each time it’s offered. One respondent said, “Definitely resources because we all have to meet our revenue for the year, so this program being so long, we have to try to get some grants and things like that. I know that we’re hoping to run it for a long time and see if there’s interest in it. I won’t really know until after the first one starting in January, if we even have interest in that program.”

Following the initial 12-week implementation, each of the study sites elected to continue offering the program, identifying it as an important program for their community. Due to price structuring within the local Parks and Recreation system, one site is required to charge for the class. They are seeking funding from local partners to eliminate charging participants and consider this a potential barrier to enrollment. Another site recommended shortening the program to 8 weeks, given the typical seasonal programming and schedules they coordinate. The study team continues to assess their implementation using the fidelity checklist for video-recorded sessions, but we no longer provide on-going feedback. We also developed a self-assessment of fidelity to be completed by facilitators, and compare those self-ratings to the study team’s assessment using the gold standard of video-taped sessions. After adapting the fidelity checklist tool to have a range of 1–2 (instead of 1–3 as originally developed), the study team rated videos for all three sites that have fully implemented the program without additional research support. This revealed agreement between self-assessment and the study team’s assessment resulting in high fidelity program implementation.

## Discussion

Community-based implementation scale-out research evaluates how efficacious interventions should be modified so that similar results can be achieved in different settings with more diverse populations. We report on the successful pilot implementation of an effective behavioral intervention designed to prevent and treat childhood obesity for preschool age children in community Parks and Recreation center settings. Using the RE-AIM framework to evaluate the public health impact of this pilot we identified successes and next-steps to guide the field of community-based implementation science and to inform broader implementation of the Healthier Families Program. At the organizational level, the evaluation revealed the need for close alignment between the goals of the Healthier Families program with local community priorities and community member’s interests. This alignment led to successful uptake of the program with high fidelity and attendance. Most notably, each of the Parks and Recreation centers that participated made the decision to sustain these efforts after grant funding ceased. When assessing the program for changes that affected individuals, it was evident we were able to adapt the program to reach participants from a range of socioeconomic communities. While the current study was not powered to test for changes in BMI, we demonstrated changes in the use of behavior change techniques, the use of strategies for implementing healthy behaviors and trends toward healthier nutrition, physical activity, and media use. Finally, qualitative data generated from interviews with both local Parks and Recreation staff, and program participants reiterated the successful uptake of the program in the community as well as sustained behavior change and use of the built environment.

Parks and Recreation centers around the country are prime candidates for this type of translational research, and future efforts should focus on expanding the reach of behavioral obesity interventions in environments that young children and families frequent. Fidelity monitoring, one of the most important aspects of effective replication, required multi-modal training that included ongoing feedback in response to real-time evaluation. Challenges included clearly delineating the difference between an adaptation necessary for effective contextualization in each community and how that could alter the fidelity of the program to the original evidence-based approach. An additional challenge included Parks and Recreation staff having the time to engage in training modules and to have support staff during the intervention to effectively engage both parents and children.

During the preliminary phases of this scale-out pilot, we initially engaged five community Parks and Recreation centers. Throughout the pre-assessment phases, only three elected to continue. The main reasons that sites decided not to move forward with the implementation phase were not having the necessary physical resources and not having close alignment with community participants’ priorities. Consequently, we would suggest that any attempt to replicate a community-based behavioral intervention would first conduct a careful assessment of alignment with community priorities and resources to avoid investing in a program that will have a low likelihood of success.

In this pilot scale-out implementation project, geographically diverse communities had high interest in delivering the Healthier Families program, as well as interest from families in program participation.. The pilot outcomes of healthy behavior change in parents with young children indicated similar changes over three months regardless of the community, suggesting that core skills such as setting and meeting family goals in nutrition and physical activity can be strengthened in diverse populations.

This is one of the first attempts to conduct *community*-based implementation research, especially among behavioral interventions for childhood obesity. Conceptual models, like the Consolidated Framework for Implementation Research (CFIR) provide a strong theoretical foundation [26]. The CFIR identifies five domains: inner setting, outer setting, intervention characteristics, characteristics of individuals, and processes. While the CFIR was initially designed for clinical research, these domains provide a useful framework for evaluating community-based implementation as well. The most notable trial published in dissemination and implementation research is the Diabetes Prevention Program (Y-DPP), which has been widely implemented in YMCAs across the country. Interestingly, the dissemination of the Y-DPP into traditionally minority communities has not resulted in the same behavior change as the original trial [27]. Along with our results, this points to future directions for research to investigate the causes for this drift. We would suggest the uses of conceptual models like the CFIR and evaluation models like RE-AIM to guide future research in community-based implementation research.

This study had several limitations. First, as this was designed as a pilot study, we engaged three Parks and Recreation centers and 26 families. Consequently, we cannot make generalizations about the effectiveness of the intervention, or say with certainty that these implementation methods will be applicable in other contexts. Furthermore, the study sites and participants who engaged in the program were early adopters, enrolling without use of targeted recruitment methods, and were likely to be more highly engaged than the general population. Our assessment approach for health behaviors was limited to pre-post surveys, which may be subject to either recall or social desirability bias given behaviors were not recorded in real time. Finally, the RE-AIM framework defines maintenance as six months following an intervention, however, we were only able to collect data 3 months after intervention completion due to the feasibility of maintaining follow-up in each community.

## Conclusions

To generate sustainable solutions to the childhood obesity epidemic, identifying tested strategies that are ripe for dissemination and implementation will be necessary. Yet, this is only the first step in the process. This must be followed by a systematic approach to adapt and contextualize these strategies to ensure effective spread. While our pilot program had a small sample size, our results demonstrate promise and highlight the importance of contextualizing the intervention for effective scaling-out while providing a rigorous approach to ensure high fidelity. Future research in community-based implementation science should focus on developing robust training platforms that can reproducibly train interventionists from a wide range of backgrounds to deliver health behavior interventions with high fidelity. Ensuring both practical implementation in a variety of context and maintenance of the original program’s essential components requires systematic strategies that can be utilized for effective implementation of programs that have promise to improve the public’s health.

## Additional file

Additional file 1: Example of an ADAPT Fidelity Report: Module- Healthy Snacks & Drinks. (DOCX 22 kb)

## Abbreviations

BMI: Body mass index
CFIR: Consolidated Framework for Implementation Research
DPP: Diabetes Prevention Program
IQR: inter-quartile range
RCT: Randomized controlled trial
RE-AIM: Reach, Effectiveness, Adoption, Implementation, Maintenance
