# Supplementary material for: Scale-out of a community-based behavioral intervention for childhood obesity: pilot implementation evaluation
Source: BMC Public Health. 2018 Apr 13;18:498. doi: 10.1186/s12889-018-5403-z (PMC5899408; doi:10.1186/s12889-018-5403-z)
Supplement: Supplementary file 1 — Example of an ADAPT Fidelity Report: Module- Healthy Snacks & Drinks. (DOCX 22 kb) [file 12889_2018_5403_MOESM1_ESM.docx]

**Additional file 1: Example of an ADAPT Fidelity Report: *Module- Healthy Snacks & Drinks***

|  |  | **SESSION OUTLINE** | **HAPPENED** | | | **COMMENTS** |
| --- | --- | --- | --- | --- | --- | --- |
|  |  |  | 0=did not happen  1=partially completed  2=completed | | |  |
| 1. | □ | Program leader welcomes participants. | **0** |  | **2** |  |
| 2. | □ | Reviewed last week’s goals and assessed if goals were met. | **0** |  | **2** |  |
| 3. | □ | Facilitated problem solving when striving to reach last week’s goals (if no problems, then mark as completed). | **0** |  | **2** |  |
| 4. | □ | **Didactic teaching of key message 1: Smart Snacking** | **0** | **1** | **2** |  |
| 5. | □ | **Didactic teaching of key message 2: Re-think Your Drinks** | **0** | **1** | **2** |  |
| 6. | □ | **Didactic teaching of key message 3: Be Sugar Smart** | **0** | **1** | **2** |  |
| 7. | □ | Social Media: Review how to access session booklets on the ADAPT website. | **0** |  | **2** |  |
| 8. | □ | New individual goal setting for this week’s topic. | **0** |  | **2** |  |
| 9. | □ | Program leader facilitates small group discussions (break into small groups if total participants >5). | **0** |  | **2** |  |
| 10. | □ | Program leader distributed the module booklet during the session. | **0** |  | **2** |  |
